# Sigmoid Sinus Wall Reconstruction for Venous Pulsatile Tinnitus: A Critical Review of Surgical Outcomes, Hemodynamic Evidence, and Pressure-Associated Findings

**DOI:** 10.3390/audiolres16040106

**Published:** 2026-07-27

**Authors:** Bulent Mamikoglu, Pankajavalli Ramakrishnan, Fawaz Al-Mufti, Gerard Gianoli

**Affiliations:** 1 WMCHealth Physicians Advanced ENT Services, 19 Baker Ave., Suite 301, Poughkeepsie, NY 12601, USA; bulent.mamikoglu@wmchealth.org; 2 WMCHealth Physicians Neurosurgery, 19 Baker Avenue, Suite 302, Poughkeepsie, NY 12601, USA; pankajavalli.ramakrishnan@wmchealth.org; 3 WMCHealth Brain and Spine Institute—Neurosurgery, 100 Woods Rd, ACP 4th Floor, Valhalla, NY 10595, USA; fawaz.al-mufti@wmchealth.org; 4 The Ear and Balance Institute, Covington, LA 70433, USA

**Keywords:** pulsatile tinnitus, sigmoid sinus wall anomaly, sigmoid sinus wall reconstruction, transverse sinus stenosis, idiopathic intracranial hypertension, venous sinus stenting, venous hemodynamics

## Abstract

Background/Objectives: To critically evaluate the evidence on sigmoid sinus wall reconstruction (SSWR) for venous pulsatile tinnitus (PT), with emphasis on outcome durability, objective hemodynamic assessment, pressure-associated findings, and safety. Methods: A critical review with a structured literature search and reporting informed by PRISMA 2020 was performed using PubMed/MEDLINE and backward citation searching. Embase, Scopus, and the Cochrane Library were used to identify contextual secondary reviews and to cross-check the primary-study set. A descriptive study-level appraisal addressed design, cohort size, follow-up, outcome objectivity, hemodynamic measurements, and complication reporting. The manuscript is presented as a critical review because no prospective registration or formal risk-of-bias assessment was undertaken. Results: Thirty-four records were identified, two duplicates were removed, and 32 records were screened; 10 studies were included in the qualitative synthesis. These comprised dedicated SSWR/resurfacing outcome cohorts and clinical, imaging, or pressure/hemodynamic studies, with some studies contributing to more than one domain. The cohorts were small, heterogeneous, and predominantly retrospective, and only one cohort had mean follow-up exceeding 5 years. In that cohort, complete PT elimination was reported in 24/37 ears (64.9%), with significant partial improvement in 10 additional ears, yielding an initial satisfactory response in 34/37 ears (91.9%); after recurrence was considered, the source authors reported long-term success in 31/37 ears (83.8%). IIH (3/35), papilledema (5/35), and venous sinus stenting (2/35) were reported separately during follow-up and were not combined into a composite prevalence. Objective pre- and postoperative intracranial pressure or venous hemodynamic measurements were sparse, and complication reporting was inconsistent. Conclusions: This critical review suggests symptomatic benefit from SSWR in selected patients but finds insufficient evidence to determine whether the procedure normalizes intracranial pressure, corrects upstream venous hemodynamics, prevents recurrence, or modifies long-term disease. Comparative treatment recommendations cannot be supported. Prospective multicenter studies with standardized objective, patient-reported, and safety outcomes are required.

## 1. Introduction

Sigmoid sinus wall anomalies (SSWAs), including sigmoid sinus dehiscence and diverticulum, are increasingly recognized as important structural correlates of venous pulsatile tinnitus (PT). Surgical approaches such as sigmoid sinus wall reconstruction (SSWR) and resurfacing have demonstrated favorable symptomatic outcomes in appropriately selected patients [1,2,3,4,5,6]. Despite growing surgical experience, the relationship between SSWAs and the broader physiology of intracranial venous pressure regulation remains incompletely understood [7,8,9,10,11].

Historically, SSWAs have been conceptualized primarily as localized structural or acoustic abnormalities in which focal bony dehiscence or diverticular remodeling permits transmission of venous flow-related sound to the auditory system [2]. However, multiple studies have reported recurring demographic and radiographic associations within these patient populations, including female predominance, elevated body mass index (BMI), transverse sinus stenosis (TSS), headaches, papilledema, spontaneous cerebrospinal fluid (CSF) leaks, and, in some cases, formally diagnosed idiopathic intracranial hypertension (IIH) [6,7,8,9,10,11,12,13,14,15]. These observations have raised increasing interest regarding whether several documented SSWAs may coexist with altered venous or intracranial pressure physiology rather than representing purely isolated acoustic phenomena.

The relationship between venous PT and IIH has traditionally been approached using categorical diagnostic frameworks in which patients either fulfill formal IIH criteria or are considered independent of pressure-related disease [12]. However, clinical and radiographic studies have described overlap among venous PT, SSWAs, IIH, and TSS [7,8,9,10,11,12,13,14,15]. Within such a framework, papilledema, venous sinus stenosis, elevated trans-stenotic pressure gradients, spontaneous CSF leak syndromes, headache syndromes, and venous PT may represent overlapping manifestations in selected patients; the available studies do not establish that these findings share a single causal pathway.

Recent surgical outcome studies have provided valuable insight into tinnitus improvement following SSWR or resurfacing [1,2,3,4,5,6]. Most published studies, however, have focused primarily on symptomatic outcomes rather than broader venous or intracranial pressure-associated findings, and only one currently available cohort provides mean follow-up beyond 5 years [1].

Three prior systematic reviews summarized surgical or endovascular treatment of sigmoid sinus-associated PT but were not eligible as primary cohorts in the present extraction because they represent secondary evidence. Wang et al. synthesized 139 patients with a mean follow-up of 21.1 months [16]. Liu et al. reviewed 121 sigmoid sinus resurfacing cases, reporting 75% complete and 10% partial resolution and major complications in 4% of cases [17]. Sathya et al. compared endovascular and surgical treatment of sigmoid sinus diverticulum across predominantly case reports and small series [18]. The present review differs by focusing on outcome durability beyond 5 years, the use of objective hemodynamic or intracranial pressure measurements, delayed pressure-associated findings, and the limitations of safety reporting.

The purpose of this review was to critically appraise the SSWR and SSWA surgical literature, with particular attention to the durability and objectivity of reported outcomes, the extent of hemodynamic assessment, pressure-associated findings, and procedure-related safety. We also sought to identify whether the available evidence can support conclusions regarding intracranial pressure, venous hemodynamics, long-term disease modification, or comparative treatment selection.

## 2. Materials and Methods

### 2.1. Literature Search Strategy

This critical review used a structured literature search and descriptive qualitative synthesis. The search and reporting were informed by PRISMA 2020. The manuscript is presented as a critical review because no prospective registration or formal risk-of-bias assessment was undertaken. The primary search for eligible original studies was performed in PubMed/MEDLINE and supplemented by backward citation searching of included articles. PubMed/MEDLINE was selected because this narrow topic is concentrated in otology, neurotology, and neuroradiology journals indexed in MEDLINE. Embase, Scopus, and the Cochrane Library were used to identify relevant secondary reviews and to cross-check the primary-study set, but they were not treated as independent primary-search databases. Accordingly, the flow diagram reports only PubMed/MEDLINE and citation-search yields. Search terms included combinations of:

- “sigmoid sinus wall anomaly,”
- “sigmoid sinus dehiscence,”
- “sigmoid sinus diverticulum,”
- “sigmoid sinus wall reconstruction,”
- “sigmoid sinus resurfacing,”
- “venous pulsatile tinnitus,”
- “transverse sinus stenosis,”
- “venous sinus stenosis,”
- and “idiopathic intracranial hypertension.”

The literature search was last updated in May 2026. The PubMed/MEDLINE strategy was: ((“sigmoid sinus wall anomaly” OR “sigmoid sinus dehiscence” OR “sigmoid sinus diverticulum”) AND (“sigmoid sinus wall reconstruction” OR “sigmoid sinus resurfacing” OR surgery) AND (“pulsatile tinnitus” OR “venous pulsatile tinnitus”)) OR ((“transverse sinus stenosis” OR “venous sinus stenosis” OR “idiopathic intracranial hypertension”) AND “pulsatile tinnitus”).

Primary studies were included if they reported SSWR or related surgical outcomes for SSWAs, described associated venous sinus abnormalities, or provided objective pressure or hemodynamic information relevant to venous PT. Secondary systematic reviews were excluded from the primary-study extraction but retained for contextual discussion. Duplicate reports, isolated case reports without meaningful outcome data, and non-English publications were excluded. Initial title and abstract screening was performed by B.M.; full-text eligibility decisions and extracted study-level data were checked by G.G., with disagreements resolved by discussion among the authors.

Because the included literature consisted predominantly of heterogeneous retrospective surgical case series with variable outcome reporting, the manuscript was classified as a critical review with a structured search and descriptive qualitative synthesis. Formal quantitative meta-analysis was not performed because of substantial heterogeneity in study design, follow-up duration, outcome definitions, and reporting methodology. The review was not prospectively registered.

**Figure 1:**
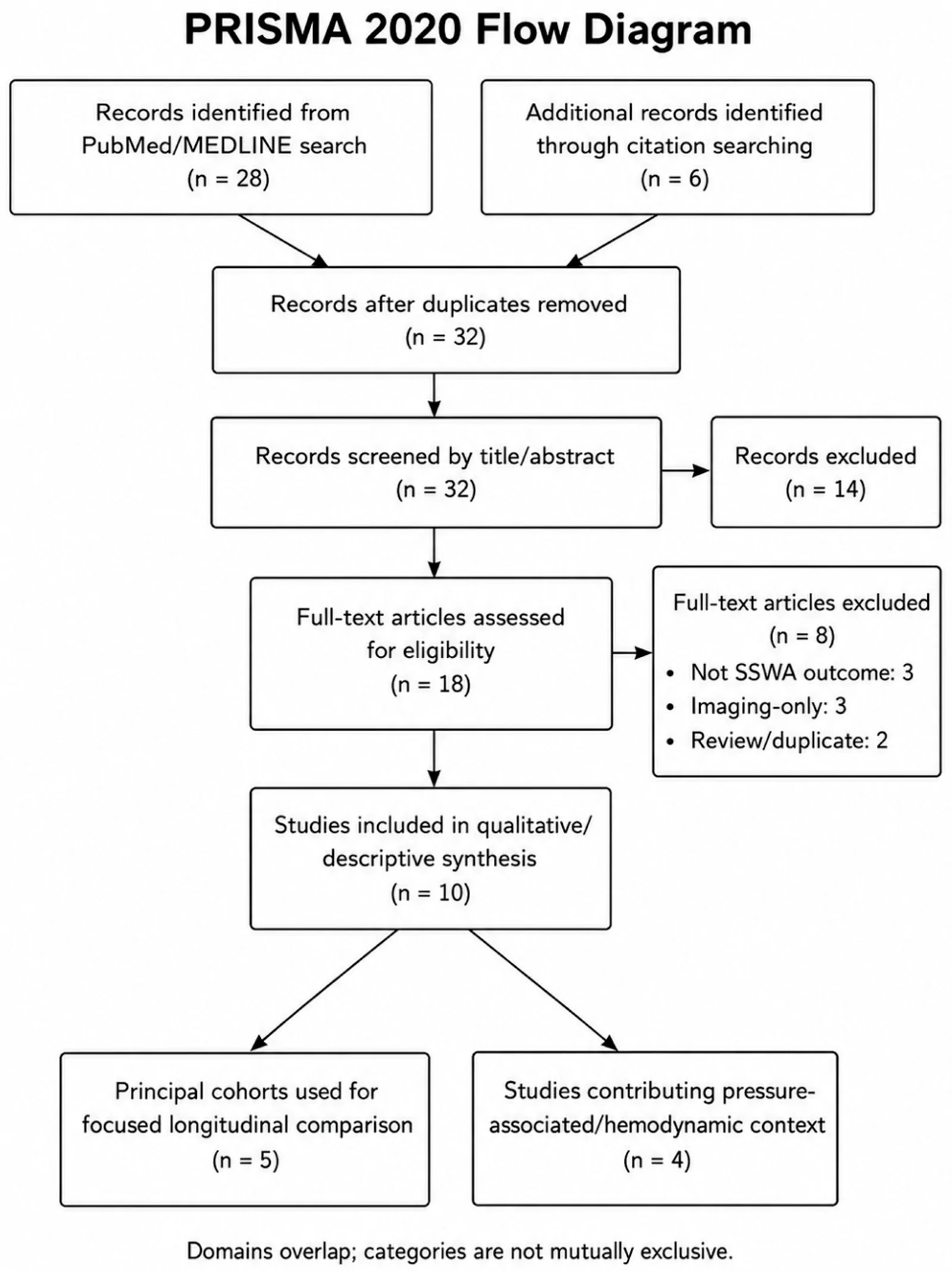
PRISMA-style flow diagram documenting the structured search used for this critical review. Thirty-four records were identified (28 from PubMed/MEDLINE and 6 through citation searching), two duplicates were removed, and 32 records were screened. The two boxes branching below the 10 included studies are parallel descriptive synthesis domains rather than sequential eligibility steps. The n = 5 box denotes the principal cohorts used for focused longitudinal comparison [1,2,3,4,5]; Lim et al. [6] is additionally included in Table 1 as a predictive/outcomes study. The n = 4 pressure-associated/hemodynamic context domain comprises Grewal et al. [7], Hewes et al. [8], Harvey et al. [10], and Kline et al. [11]. As defined in Section 2.3 and Section 3.5, this domain includes clinical and imaging associations as well as objective pressure assessment and does not imply that all four studies reported direct venous manometry or flow measurements. The domains overlap and are not mutually exclusive.

Study identification and screening are summarized in the PRISMA-style flow diagram (Figure 1). The final post-inclusion boxes branch from the 10 included studies and represent parallel descriptive synthesis domains rather than sequential eligibility steps. The box labeled “principal cohorts used for focused longitudinal comparison (n = 5)” comprises studies [1,2,3,4,5]; Lim et al. [6] was additionally retained as a predictive/outcomes study and is included in the comprehensive study-characteristics table. The pressure-associated/hemodynamic context domain (n = 4) comprises Grewal et al. [7], Hewes et al. [8], Harvey et al. [10], and Kline et al. [11]. These domains overlap and are not mutually exclusive.

### 2.2. Literature Characteristics

The available literature consisted predominantly of retrospective surgical case series, institutional cohorts, and technical reports. Most studies focused primarily on symptomatic tinnitus outcomes and provided limited patient-level detail regarding associated venous pressure-related findings. Only one currently available cohort study reported mean postoperative follow-up exceeding 5 years following SSWR [1].

### 2.3. Data Extraction and Descriptive Analysis

Published studies were reviewed for:

- cohort size,
- duration of follow-up,
- demographic characteristics,
- headache prevalence,
- formally diagnosed IIH,
- venous sinus stenting,
- CSF diversion procedures,
- spontaneous CSF leak repair,
- papilledema, including diagnostic basis and timing when reported,
- and long-term pulsatile tinnitus outcomes.

Particular attention was given to the long-term cohort reported by Ahanotu et al. [1], from which detailed pressure-associated findings and long-term outcome data were extracted. Papilledema was added as an extraction variable during iterative full-text review after it was identified in the long-term cohort rather than being specified in a prospectively registered protocol. Where reported, the diagnostic basis and timing of papilledema were recorded; Ahanotu et al. identified papilledema as an ophthalmologist-diagnosed finding during follow-up, although the exact timing and baseline ophthalmologic status were not available.

For synthesis-domain classification, “pressure-associated/hemodynamic context” was defined broadly as reporting at least one of the following: venous sinus anatomy or stenosis, a clinical association with an intracranial-pressure phenotype, measured intracranial pressure, venous manometry, or quantitative flow assessment. This domain label did not imply that every study contained direct physiological measurements. Objective hemodynamic evidence was considered separately and was limited to measured intracranial pressure, trans-stenotic venous pressure gradients, or quantitative venous flow data.

Because patient-level overlap between reported categories was unavailable in most studies, findings were analyzed descriptively. No inferential statistical comparisons were performed, and all observations should be interpreted as hypothesis-generating rather than establishing causal relationships.

### 2.4. Evidence Quality and Safety Appraisal

Each included study was appraised descriptively across study design, cohort size, follow-up duration, selection criteria, outcome definition, reliance on patient-reported versus objective measurements, reporting of venous manometry or intracranial pressure, and complication reporting. Because this manuscript is presented as a critical review rather than a formal systematic review, a formal study-level risk-of-bias checklist and pooled numerical quality score were not applied. The descriptive appraisal was used to qualify the strength of each conclusion, and the absence of a formal risk-of-bias assessment remains an explicit limitation.

## 3. Results

### 3.1. Overview of Published SSWR Cohorts

The search identified 34 records (28 from PubMed/MEDLINE and 6 through citation searching). After removal of two duplicates, 32 records were screened, and 10 studies were included in the qualitative synthesis. The included set comprised six SSWR/resurfacing outcome studies [1,2,3,4,5,6]. Five of these were the principal cohorts used for the focused longitudinal comparison [1,2,3,4,5], whereas Lim et al. [6] was retained as a predictive/outcomes study because it evaluated resurfacing outcomes together with the water occlusion test. The remaining included studies comprised two clinical or imaging phenotype studies [7,10], one study focused on the pattern and severity of transverse sinus stenosis in patients with SSWA [8], and one prospective IIH/SSWA study [11]. Some studies contributed to more than one synthesis domain. The evidence base consisted predominantly of small, retrospective, single-center cohorts with heterogeneous selection criteria, surgical techniques, outcome definitions, and follow-up durations.

The dedicated surgical cohorts were small, generally including 13–35 patients, and most reported short- or medium-term follow-up. Lim et al. [6], which evaluated resurfacing outcomes and the predictive value of the water occlusion test, is included explicitly in Table 1. Detailed patient-level reporting of pressure-associated findings, objective physiology, and adverse events was limited. Ahanotu et al. [1] was the only identified SSWR cohort with mean follow-up exceeding 5 years (9.51 years). All 10 included studies are summarized in Table 1, which separately reports study design and sample, synthesis domain(s), intervention or exposure, follow-up, key extracted findings, and the principal limitation for each study.

### 3.2. Outcomes in the Only Cohort with Mean Follow-Up Exceeding 5 Years

The cohort reported by Ahanotu et al. [1] included 35 patients and 37 operated ears with a mean postoperative follow-up of 9.51 years. The study population was predominantly female (94%), with a mean BMI of 34 kg/m^2^. Headaches were reported in 54.3% of patients.

Complete elimination of PT was reported in 24 of 37 operated ears (64.9%), and 10 additional ears demonstrated significant partial improvement, yielding an initial satisfactory response in 34 of 37 ears (91.9%). Four patients subsequently reported recurrent symptoms. After recurrence was considered and one contralateral recurrence was not classified as failure of the operated ear, the source authors reported recurrence-adjusted long-term success in 31 of 37 ears (83.8%) [1]. These observations suggest persistence of symptomatic benefit in this cohort, but a single retrospective series cannot establish generalizable long-term efficacy or physiological disease modification.

### 3.3. Pressure-Associated Findings in the Long-Term Cohort

During delayed postoperative follow-up, three patients (8.6%) were reported to have developed or received a formal diagnosis of IIH [1]. Within this subgroup:

- one patient underwent ventriculoperitoneal (VP) shunt placement,
- one underwent spontaneous cerebrospinal fluid (CSF) leak repair,
- and one was managed medically for IIH-related disease.

The published source also explicitly reported ophthalmologist-diagnosed papilledema in five patients (14.3%) in both its Results and Table 2, and transverse sinus venous stent placement in two patients (5.7%) [1]. These findings were ascertained during postoperative follow-up, but the exact timing, baseline ophthalmologic examination, and baseline manometric status were not sufficiently reported to determine whether they were pre-existing, newly developed, or unrelated to the preceding SSWR.

### 3.4. Pressure-Associated Clinical Phenotypes Across Cohorts

Across the reviewed literature, demographic features, symptoms, objective signs, diagnoses, imaging findings, and interventions were reported inconsistently and at different time points. These included female predominance, transverse sinus abnormalities, headaches, papilledema, IIH, and venous interventions [1,3,4]. Because these variables are conceptually and temporally distinct, they were summarized separately and were not combined into a composite prevalence. Their occurrence supports consideration of a broader hemodynamic context in selected patients but does not establish a shared causal pathway.

### 3.5. Evidence Quality, Objective Physiological Outcomes, and Safety Reporting

Across the included studies, treatment outcomes were dominated by patient-reported tinnitus improvement, with variable definitions of complete, partial, satisfactory, recurrent, and persistent symptoms. Blinding, control groups, and validated standardized PT outcome instruments were generally absent. These features increase susceptibility to recall bias, observer bias, placebo effects, and reporting variability. Four studies were categorized as contributing pressure-associated/hemodynamic context: Grewal et al. [7], Hewes et al. [8], Harvey et al. [10], and Kline et al. [11]. This operational category was intentionally broader than direct hemodynamic measurement. Grewal et al. [7] provided clinical and CT associations between sigmoid sinus diverticulum/dehiscence and PT; Harvey et al. [10] described the clinical and epidemiologic relationship between sigmoid sinus wall anomalies and the IIH phenotype; Hewes et al. [8] characterized the imaging distribution and severity of transverse sinus stenosis in patients with SSWA; and Kline et al. [11] prospectively evaluated CT-detected SSWA and lumbar puncture opening pressure in patients with IIH with and without PT. Of these four studies, only Kline et al. [11] included an objective intracranial-pressure measurement. None reported quantitative venous flow measurements, trans-stenotic venous manometry linked to SSWR, or paired pre- and postoperative physiological measurements sufficient to show that SSWR normalized intracranial pressure or venous hemodynamics. Adverse events were also inconsistently defined and reported, preventing calculation of a reliable pooled complication rate.

## 4. Discussion

This critical review identifies a clinically relevant but methodologically weak evidence base. Although SSWR cohorts generally report symptomatic improvement in selected patients with venous PT, the literature is composed largely of small retrospective series, heterogeneous procedures, inconsistent outcome definitions, and predominantly subjective endpoints. Only one identified SSWR cohort had mean follow-up exceeding 5 years, and dedicated objective pressure or hemodynamic assessment was uncommon. The findings therefore support a cautious appraisal of symptomatic outcomes rather than conclusions about intracranial pressure, venous physiology, or long-term disease modification.

The available studies suggest that SSWR may reduce PT symptoms in appropriately selected patients [1,2,3,4,5]. In the Ahanotu et al. cohort, complete PT elimination was reported in 64.9% of operated ears, initial complete-or-significant-partial improvement in 91.9%, and recurrence-adjusted long-term success in 83.8% [1]. These distinct outcome definitions should not be conflated. The results derive from a retrospective series without a control group and with outcomes based primarily on symptom reporting; they therefore do not establish physiological efficacy or a generalizable treatment effect for all patients with SSWAs.

IIH, papilledema, venous sinus stenting, CSF diversion, and spontaneous CSF leak repair were reported during postoperative follow-up in the Ahanotu cohort [1]. Their chronology does not establish that these conditions were present before surgery, caused the original wall anomaly, or resulted from SSWR. Baseline testing, patient-level overlap, timing of onset, and objective pre- and postoperative physiological measurements were insufficient for causal inference.

A central limitation of the literature is the mismatch between the proposed hemodynamic mechanisms and the outcomes actually measured. Most studies assessed symptom change without standardized venous manometry, intracranial pressure assessment, quantitative flow imaging, or longitudinal ophthalmologic evaluation. Subjective improvement is clinically meaningful, but it cannot establish correction of upstream venous flow, normalization of intracranial pressure, or prevention of future pressure-associated disease.

Venous sinus stenting series have reported high rates of PT resolution in selected patients with hemodynamically significant venous sinus stenosis [19]. These data cannot be compared directly with SSWR cohorts because treatment indications, anatomy, selection criteria, outcome definitions, antiplatelet exposure, and follow-up differ substantially. Table 3 is therefore presented only as a noncomparative conceptual overview of the physiological targets and evidentiary limitations of the two procedures, not as an efficacy comparison or treatment recommendation.

SSWR is intended to modify the local acoustic transmission interface, whereas venous sinus stenting is intended to reduce a measured venous outflow obstruction. Whether either intervention modifies broader intracranial pressure physiology must be demonstrated with procedure-specific objective measurements rather than inferred from symptom improvement alone.

### 4.1. Potential Hemodynamic Mechanisms

Several hemodynamic mechanisms have been proposed to link transverse sinus stenosis, venous PT, and sigmoid wall remodeling. TSS can be associated with accelerated jet flow, altered pulsatility, vortical flow, and trans-stenotic pressure gradients within the transverse-sigmoid system [7,8,9,12,13,14,15]. These mechanisms are biologically plausible, but the reviewed SSWR studies did not provide sufficient longitudinal physiological measurements to establish that abnormal wall stress causes SSWAs or that reconstruction reverses the upstream hemodynamic process.

**Figure 2:**
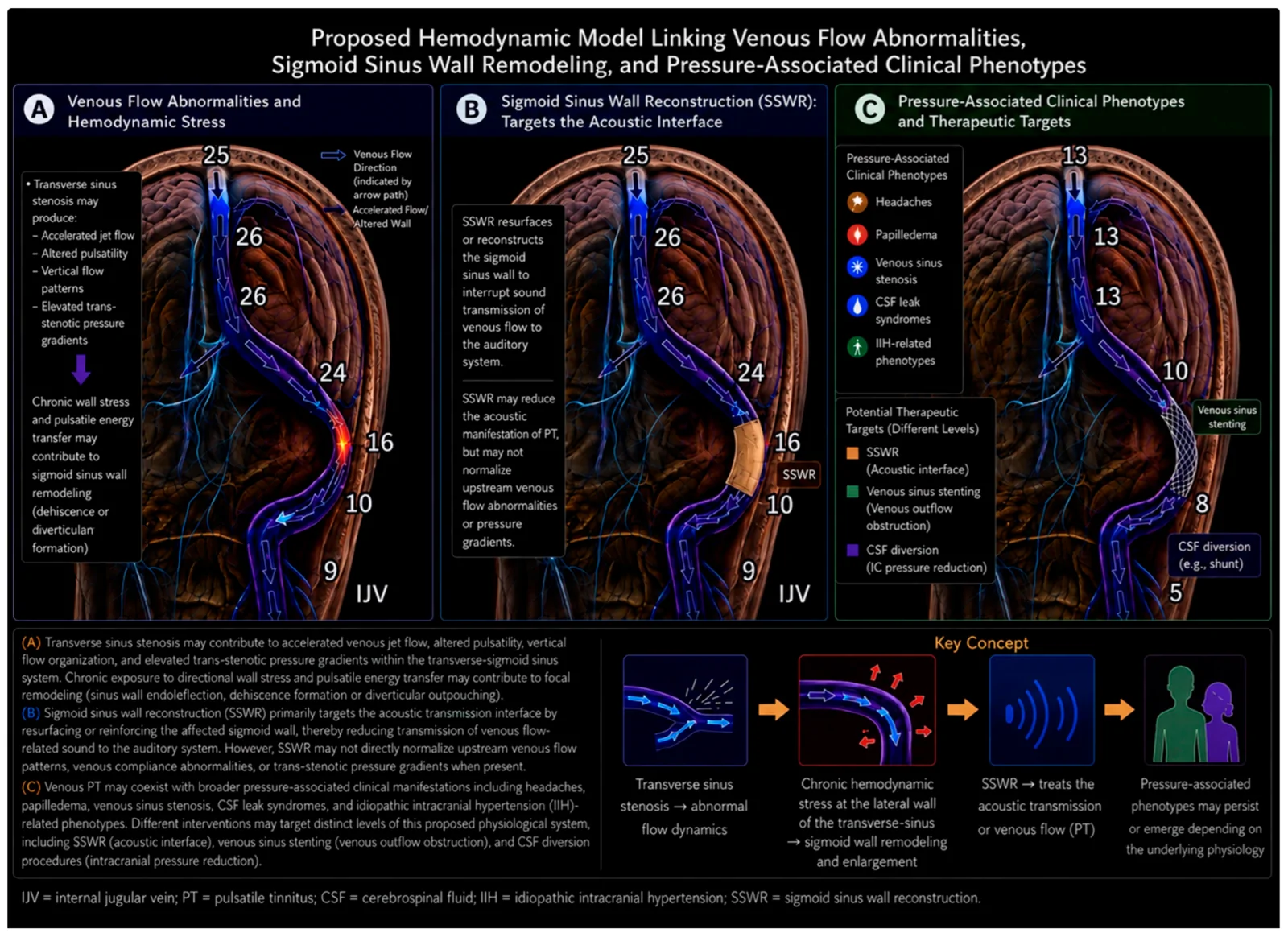
Author-created, AI-assisted conceptual schematic of the proposed relationship among venous pressure gradients, the local sigmoid wall acoustic interface, and interventions directed at different physiological levels. The anatomical base illustration is original artwork created specifically for this manuscript and was not reproduced or adapted from a third-party publication; therefore, no external copyright permission was required. IMPORTANT: the numerical labels are hypothetical values used solely to illustrate directional pressure differences. They are not patient-level measurements, pooled estimates, or values extracted from any included study. Panel (**A**) illustrates a trans-stenotic gradient and disturbed flow; Panel (**B**) illustrates SSWR at the local acoustic interface without implying normalization of upstream pressure; Panel (**C**) illustrates lower hypothetical pressure values after an upstream venous intervention and distinguishes venous stenting from CSF diversion. AI-assisted graphic tools were used for rendering, layout, labeling, and visual integration under author direction. The figure is hypothesis-generating and is not a head-to-head treatment comparison.

The conceptual model in Figure 2 should therefore be interpreted as hypothesis-generating. SSWR may reduce transmission of venous sound by modifying the local wall interface, but the available studies do not establish its effect on intracranial pressure, venous compliance, flow organization, or trans-stenotic gradients. All numerical labels within the figure are hypothetical values used only to illustrate directional pressure differences; they are not patient-level measurements, pooled data, or values extracted from any included study.

Venous sinus stenting and CSF diversion act at different anatomical and physiological levels, but comparative treatment conclusions cannot be drawn from the current evidence. Future mechanistic studies should pair symptom outcomes with quantitative flow imaging, venous manometry, intracranial pressure assessment when clinically indicated, and longitudinal imaging. The proposed relationships are summarized in Figure 2.

### 4.2. Clinical Implications

The current evidence is insufficient to define a universal diagnostic or treatment algorithm for patients with venous PT and SSWAs. Broader evaluation for ophthalmologic or venous pressure-related disease may be clinically appropriate when supported by independent findings such as visual symptoms, papilledema, spontaneous CSF leak, or radiographically significant venous stenosis; however, this recommendation derives from established clinical assessment principles rather than comparative evidence from the reviewed SSWR cohorts.

Not all patients with SSWAs have IIH, require lumbar puncture or venous manometry, or are candidates for venous sinus stenting. Treatment selection should remain individualized, and the present review should not be used to infer superiority of SSWR, stenting, CSF diversion, or medical therapy.

### 4.3. Reported Complications and Limitations of Safety Assessment

Safety reporting was incomplete and nonuniform across the included studies. Adverse events were not consistently defined, actively ascertained, graded, or reported with clear denominators and follow-up intervals; therefore, a pooled complication rate could not be calculated. Potential complications of SSWR include injury to the venous sinus, bleeding, thrombosis or outflow compromise, wound or infectious complications, hearing change, persistent or recurrent PT, and revision surgery. Venous sinus stenting carries a different risk profile that may include access-site complications, intracranial hemorrhage, venous thrombosis, in-stent or adjacent-segment restenosis, and complications related to antiplatelet therapy [1,2,3,4,5,6,13,14,15,19]. The small retrospective cohorts and incomplete adverse-event reporting prevent reliable comparison of procedural safety. Absence of reported complications in these series should not be interpreted as evidence of negligible morbidity.

### 4.4. Limitations

Several limitations substantially constrain interpretation. Only 10 studies entered the qualitative synthesis, and the cohorts were small, predominantly retrospective, and heterogeneous in patient selection, operative technique, outcome definitions, follow-up duration, and reporting methods. Most outcomes were subjective and susceptible to recall, observer, placebo, and reporting effects. Control groups, blinded assessment, standardized PT instruments, objective pre- and postoperative physiological measurements, and systematic adverse-event surveillance were generally absent. Patient-level overlap and chronology among IIH, papilledema, stenting, and other findings were unavailable, so no composite prevalence was calculated. The primary original-study search was limited to PubMed/MEDLINE with backward citation searching. Although secondary reviews were cross-checked in Embase, Scopus, and the Cochrane Library, the absence of a full independent multi-database primary search creates a risk that eligible studies using nonstandard terminology were missed. Initial screening was performed by one author with full-text eligibility and extraction checked by the corresponding author; the review was not prospectively registered and, consistent with its reclassification as a critical review, did not use a formal validated risk-of-bias instrument. No causal relationship between venous pressure abnormalities and SSWAs can be established, and the evidence is insufficient for comparative treatment recommendations or clinical decision rules.

### 4.5. Future Directions

The most informative next step would be prospective, preferably multicenter, studies using a prespecified core outcome set that includes:

- validated patient-reported PT severity and quality-of-life measures with explicit definitions of complete, partial, recurrent, and persistent symptoms;
- standardized audiologic and objective sound-assessment methods when feasible;
- ophthalmologic evaluation and intracranial pressure assessment when clinically indicated;
- venous manometry and quantitative flow imaging before and after intervention;
- computational flow modeling and longitudinal structural imaging;
- systematic, graded reporting of perioperative and delayed complications, revisions, and antiplatelet-related events;
- prospective multicenter enrollment with follow-up extending beyond 5 years; and
- separately designed comparative studies of SSWR, venous sinus stenting, and pressure-lowering therapy in patients meeting clearly defined treatment criteria.

Such studies are required before the field can determine whether SSWR changes only the acoustic manifestation of PT or also modifies venous or intracranial pressure physiology, and before valid treatment comparisons can be made.

## 5. Conclusions

This critical review suggests that SSWR can provide meaningful symptomatic improvement in selected patients with venous PT. However, the evidence is derived predominantly from small, retrospective, single-center cohorts with heterogeneous procedures, outcome definitions, follow-up durations, and incomplete safety reporting. Only one identified SSWR study had mean follow-up exceeding 5 years, and most outcomes were subjective rather than physiological.

Current evidence is insufficient to determine whether SSWR normalizes intracranial pressure, corrects upstream venous hemodynamics, prevents recurrence, or modifies the underlying disease process. It is also insufficient to support direct comparisons or treatment-selection recommendations among SSWR, venous sinus stenting, CSF diversion, and medical therapy.

Until prospective multicenter evidence with standardized objective, patient-reported, and safety outcomes is available, SSWR should be regarded primarily as a procedure directed toward the local acoustic manifestation of venous PT rather than an established treatment for broader intracranial pressure or venous hemodynamic disease.

## Figures and Tables

**Table 1:** Characteristics, Synthesis Domains, Key Extracted Findings, and Principal Limitations of All 10 Studies Included in the Critical Review.

Study	Design and Sample	Synthesis Domain(s)	Intervention/Exposure	Follow-Up	Key Extracted Findings	Principal Limitation
Ahanotu et al. [1] (2024)	Retrospective long-term cohort; 35 patients/37 ears	Long-term SSWR outcomes; delayed pressure-associated findings; safety	SSWR	Mean 9.51 years	Complete 24/37; partial 10/37; initial satisfactory 34/37; recurrence-adjusted success 31/37; IIH 3/35, papilledema 5/35, stenting 2/35 during follow-up	Single-center retrospective cohort; chronology, baseline physiology, category overlap, and adverse-event ascertainment limited
Eisenman [2] (2011)	Retrospective case series; 13 patients/14 ears	SSWR outcomes; safety	Transmastoid SSWR	Variable; mean NR	Complete PT resolution in 14/14 ears; 2 major postoperative complications without permanent morbidity	Small uncontrolled technical series; follow-up and objective physiology incompletely reported
Zeng et al. [3] (2016)	Retrospective cohort; 34 patients, including 27 surgical patients	SSWR outcomes; safety	SSWR	At least 25 months; mean 44.96 months in surgical cohort	Complete 14/27; partial 5/27; unchanged 7/27; worse 1/27; no severe complications reported	Single-center nonrandomized study; outcomes predominantly subjective
Lee et al. [4] (2020)	Retrospective cohort; 20 patients	Surgical outcomes; psychoacoustic assessment; imaging	Sigmoid sinus surgery	Median 37 months (range 12–54)	18/20 female; mean BMI 23.9 ± 3.7 kg/m^2^; TSS in 4/15 assessed; postoperative subjective and psychoacoustic improvement	Small cohort; outcome definitions and objective physiological assessment were limited
Ettyreddy et al. [5] (2021)	Retrospective cohort; 19 patients	Resurfacing outcomes; audiology; safety	Transmastoid sigmoid resurfacing	NR	Complete resolution 16/19; partial response 2/19; no improvement 1/19; no significant complications; low-frequency PTA improved	Small cohort; duration of follow-up and pressure-related assessment incompletely reported
Lim et al. [6] (2023)	Retrospective cohort; 26 patients	Resurfacing outcomes; diagnostic prediction	Sigmoid sinus resurfacing; water occlusion test	Duration NR in abstracted data	Complete cure 14/26; significant improvement 7/26; stable 5/26; no severe complications reported	Small retrospective cohort; no standardized ICP or venous manometry; follow-up duration not consistently reported
Grewal et al. [7] (2014)	Retrospective CT/chart study; 200 CT scans and 61 PT patients; 15 PT patients with CT-confirmed SSDD	Clinical/CT association; no direct pressure or flow measurement	Imaging/clinical assessment	Cross-sectional	SSDD was associated with PT; all 15 PT+SSDD patients were female; mean BMI 32.2 kg/m^2^	Not an intervention-outcome study; no longitudinal physiological assessment
Hewes et al. [8] (2020)	Retrospective imaging study; 36 surgical patients, 26 with adequate imaging	Imaging-defined TSS/venous outflow context; no direct pressure or flow measurement	Preoperative imaging in SSWA patients	Cross-sectional imaging analysis	Bilateral/distal TSS was common; ipsilateral distal TSS occurred in 16/16 diverticula versus 4/10 dehiscences	No direct pre/post manometry or ICP measurements; historical comparator design
Harvey et al. [10] (2014)	Retrospective cohort; 33 patients with SSWA	Clinical phenotype; IIH context; SSWR outcomes; no direct hemodynamic measurement	Transmastoid SSWR	NR	13 diverticula and 20 dehiscences; 30 ears responded and 3 did not; marked female predominance and elevated BMI	Retrospective institutional cohort; possible overlap with related reports; no standardized manometry or ICP assessment
Kline et al. [11] (2020)	Prospective IIH cohort; 22 enrolled, 14 underwent CT	IIH/PT/SSWA association; objective lumbar puncture opening pressure	CT and clinical/LP assessment	Cross-sectional	SSWA incidence was 70% in IIH subjects with PT versus 0% without PT; opening pressures did not differ between groups	Very small imaged sample; no surgical outcomes; association does not establish causality

Table note: NR, not reported in the extracted source data; ICP, intracranial pressure; IIH, idiopathic intracranial hypertension; SSWA, sigmoid sinus wall anomaly; SSWR, sigmoid sinus wall reconstruction; TSS, transverse sinus stenosis. The table lists every study included in the qualitative synthesis and identifies the domain(s) to which each contributed. The “Key extracted findings” and “Principal limitation” columns summarize the principal study-level observations and the main methodological constraint relevant to this critical review. Study populations may overlap across reports from the same institution; therefore, cohorts were not pooled. Ahanotu et al. is the only identified SSWR cohort with mean follow-up exceeding 5 years.

**Table 2:** Pressure-Associated Findings in the Ahanotu et al. Cohort.

Pressure-Associated Category	Reported Finding	Patients (n = 35)	Percentage	Interpretation
Formal IIH diagnosis	Developed during delayed postoperative follow-up	3	8.6%	Postoperative diagnosis; baseline status uncertain
VP shunt placement	Occurred within the IIH subgroup	1	-	Nested intervention within the IIH subgroup
Spontaneous CSF leak repair	Occurred within the IIH subgroup	1	-	Nested comorbidity/intervention within the IIH subgroup
Papilledema	Reported during follow-up; baseline status not available	5	14.3%	Objective ophthalmologic finding; chronology incompletely reported
Venous sinus stenting	Transverse sinus stent placement during follow-up	2	5.7%	Venous intervention; not a disease-prevalence category
Headaches	Reported symptom	19	54.3%	Nonspecific patient-reported symptom

Table note: Rows are non-mutually exclusive and must not be summed. VP shunt placement and CSF leak repair occurred within the IIH subgroup and are nested rather than independent categories. Diagnoses, signs, symptoms, imaging findings, and interventions are reported separately; no composite prevalence was calculated.

**Table 3:** Noncomparative Conceptual Overview of SSWR and Venous Sinus Stenting.

Feature	Sigmoid Sinus Wall Reconstruction (SSWR)	Venous Sinus Stenting
Primary target	Local acoustic transmission interface	Hemodynamically significant venous outflow obstruction
Main procedural mechanism	Resurfacing or reinforcement of the sigmoid wall	Expansion of a stenotic venous segment and reduction of the trans-stenotic gradient
Objective physiological assessment in the literature	Rarely measured directly before and after SSWR	Venous manometry commonly used for selection; post-treatment physiological reporting remains variable
Typical study population	Patients selected for sigmoid wall dehiscence or diverticulum	Patients selected for venous sinus stenosis and a clinically relevant pressure gradient
Long-term evidence	One identified cohort with mean follow-up >5 years	Limited long-term cohorts in distinct, highly selected populations
Safety considerations	Potential sinus injury, bleeding, thrombosis or occlusion, wound complications, hearing change, persistence or recurrence; reporting inconsistent	Potential access-site, hemorrhagic, thrombotic, restenosis, and antiplatelet-related complications; reporting varies
Interpretive limitation	Symptom improvement does not establish normalization of ICP or venous hemodynamics	Results cannot be directly compared with SSWR cohorts or generalized to patients without stent-selection criteria

Table note: This table summarizes conceptual targets and reporting limitations only. It is not a direct comparative effectiveness or safety analysis.

## Data Availability

No new patient-level data were generated. Study-level data extracted from the published literature are summarized in Table 1 and Table 2. Table 3 is a conceptual, noncomparative overview developed by the authors and does not represent extracted study-level data. The search terms and PubMed/MEDLINE strategy are reported in Section 2.1. Additional extraction details are available from the corresponding author upon reasonable request.
